# Girl or boy? Prenatal lead, cadmium and mercury exposure and the secondary sex ratio in the ALSPAC study

**DOI:** 10.1016/j.reprotox.2014.03.011

**Published:** 2014-07

**Authors:** C.M. Taylor, J. Golding, A.M. Emond

**Affiliations:** Centre for Child and Adolescent Health, School of Social and Community Medicine, University of Bristol, UK

**Keywords:** ALSPAC, Avon Longitudinal Study of Parents and Children, Lead, Cadmium, Mercury, Secondary sex ratio, ALSPAC

## Abstract

The aim of this study was to evaluate the effect of prenatal exposure to lead, cadmium and mercury levels on the secondary sex ratio. Whole blood samples were collected from pregnant women enrolled in the Avon Longitudinal Study of Parents and Children (ALSPAC) study at a median gestational age of 11 weeks and were analyzed for lead, cadmium and mercury. Regression analysis was used to identify associations between maternal lead, cadmium and mercury levels and the secondary sex ratio with adjustment for confounders. There was no evidence for associations between maternal lead, cadmium or mercury levels and the secondary sex ratio in this sample. It appears unlikely that alterations in the secondary sex ratio are influenced by exposure to heavy metals, but further work should be done in large cohorts in other countries to confirm these findings.

## Highlights

• The secondary sex ratio is declining in many countries.
• We evaluated the effect of prenatal exposure to heavy metals.
• There were no associations between heavy metals and the secondary sex ratio.
• Further work should be done in large cohorts in other countries.

## 1 Introduction

Many countries have seen a decline in the relative number of male infants born in recent years [1–4]. This decline could reflect a differential loss of male fetuses from spontaneous abortions: exposure to environmental stressors during particular ‘windows’ of embryonic development can undermine the successful implantation of the embryo [5] and male fetuses are known to be more vulnerable to the effects of stressors in utero. These stressors could include, for example maternal smoking [6], sex-related gene methylation differences [7] and/or sex-related differences in the hormonal milieu. Environmental chemicals acting as endocrine disruptors, such as dioxins, polychlorinated biphenyls and hexacholorbenzene, may also play a role in the secondary sex ratio [8–10]. Lead (Pb), cadmium (Cd) and mercury (Hg) can function as endocrine disruptors as Pb and Hg have been shown to have anti-oestrogenic effects, whereas Cd seems to have both oestrogenic and androgenic effects [11–14].

Pb in particular is a well known reproductive toxicant, with exposure being associated with adverse effects on sperm counts and morphology in men [15,16] and an increased rate of spontaneous abortion in women [17,18]. The specific effects of maternal Pb exposure on the secondary sex ratio have been little studied, but a recent study on a large cohort of women in Mexico found no consistent association between several measures of maternal Pb and the secondary sex ratio [19]. There have been no studies on the association of fetal exposure to Cd on the secondary sex ratio to our knowledge. With regard to Hg, evidence is limited to a study on exposure to methylmercury pollution in the 1950s, which resulted in an excess of female offspring from affected mothers resident in Minamata City in Japan, largely through consumption of contaminated fish [20].

The aim of our study was to determine the effect of maternal blood Pb, Cd and Hg levels on the secondary sex ratio in a large cohort of mother–infant pairs in the Avon Longitudinal Study of Parents and Children (ALSPAC) in the UK.

## 2 Methods

### 2.1 The ALSPAC study

The study sample was derived from the ALSPAC study, a population-based study investigating environmental and genetic influences on the health, behavior and development of children. All pregnant women in the former Avon Health Authority with an expected delivery date between 1 April 1991 and 31 December 1992 were eligible for the study; 14,541 pregnant women were initially enrolled, resulting in a cohort of 14,062 live births [21]. The social and demographic characteristics of this cohort were similar to those found in UK national census surveys [22]. Further details of ALSPAC are available at www.bris.ac.uk/alspac.

### 2.2 Ethics approval

Ethics approval for the study was obtained from the ALPAC Ethics and Law Committee and Local Research Ethics Committees.

### 2.3 Questionnaires

The mothers received four postal self-completion questionnaires during pregnancy. The questionnaires are available from the study website (http://www.bristol.ac.uk/alspac/researchers/resources-available/data-details/questionnaires/). Information collected included data on maternal and paternal age, and parity.

### 2.4 Sex of the infant

The sex of the infant was recorded at birth. If this could not be determined, for example following early pregnancy loss, then the mother was excluded from the analysis.

### 2.5 Collection, storage and analysis of blood samples

Whole blood samples were collected in acid-washed vacutainers (Becton and Dickinson, Oxford, UK) by midwives as early as possible in pregnancy. The median gestational age at the time of blood sampling was 11 weeks (interquartile range 9–13 weeks). Whole blood samples were stored in the original tube at 4 °C at the collection site before being transferred to the central Bristol laboratory within 1–4 days. Samples were at ambient temperature during transfer (up to 3 h). They were then stored at 4 °C until analysis.

Inductively-coupled plasma mass spectrometry in standard mode (R. Jones, Centers for Disease Control and Prevention (CDC), Bethesda, MD, USA CDC; Method 3009.1) was used to measure blood levels with appropriate quality controls [23]. The analyses were completed on 4285 women for Pb, 4286 women for Cd and 4134 women for Hg. One sample had a Pb level below the limit of detection (0.24 μg/dl), 1119 for Cd (0.20 μg/l) and one for Hg (0.24 μg/l). These samples were assigned a value of 0.7 times the lower limit of detection (limit of detection/√2 to reflect the log-normal distribution [24,25]).

### 2.6 Statistical analyses

All statistical analyses used SPSS v 18. The secondary sex ratio was compared categorically, with categories reflecting national guidelines where available [26], as follows. Categories of maternal Pb <5 or ≥5 μg/dl were chosen as 5 μg/dl is the US recommendation for the level of concern in pregnancy [27,28]. Categories of maternal Cd <1 or ≥1 μg/l were chosen as 1 μg/l is the only national level of concern available to our knowledge (German Federal Environmental Agency; recommendation for adults aged 18–69 years) [29]. Maternal Hg <5.8 or ≥5.8 was chosen as 5.8 μg/l is the level of concern recommended for adults by the USA on the basis that it is the blood level equivalent to the US Environmental Protection Agency's maximum oral reference dose [30]. Data on Hg levels were also analyzed according to the recommendation of Germany's Federal Environmental Agency of a level of concern of 2 μg/l [29], which is for adults aged 18–69 years old eating fish ≤3 times per month. The chi-square test was used to analyze categorical data.

Following the method of Jarrell et al. [19], maternal Pb, Cd and Hg levels were divided into quintiles. Analysis of variance was used to identify any trends across the quintiles. Adjusted logistic regression analysis was used to identify any trend in the odds ratio for being male across the quartiles of maternal Pb, Cd and Hg levels with adjustment for parity, and maternal and paternal age.

## 3 Results

The characteristics of the subsample of the ALSPAC population have been reported [23]. In brief, the subsample had slightly higher educational attainment (*p* = 0.004, chi-square test) and tended to be slightly older (*p* < 0.001, chi square test) than the rest of the ALSPAC sample, but there were no other statistically significant differences. The ALSPAC study as a whole enrolled 5538/11,008 (50.3%) male children. The percentage of boys born to mothers in the subsample analyzed here was slightly greater (2251 male/2081 female; 51.9%) than in the whole sample.

The overall secondary sex ratio was 1.08. There were no statistically significant differences in the blood levels of Pb, Cd or Hg for mothers giving birth to girls versus boys (Pb: 3.70 ± 1.49 vs 3.65 ± 1.44 μg/dl, *p* = 0.264; Cd: 0.57 ± 0.64 vs 0.58 ± 0.63 μg/l, *p* = 0.553; Hg: 2.08 ± 1.09 vs 2.06 ± 1.12 μg/l, *p* = 0.645; t tests).

There were no statistically significant differences in the secondary sex ratios for categorical analyses of the maternal levels of Pb, Cd or Hg (Table 1).

In logistic regression models adjusted for maternal and paternal age and parity there were no significant differences in the odd ratios of having a male child for quintiles 2, 3, 4 or 5 compared with quintile 1 for maternal Pb, Cd or Hg level (Table 2). There were no discernible trends in the secondary sex ratio across quintiles of maternal levels for Cd or Hg but there was slight evidence for a decline in the sex ratio in the third quintile for Pb (*p* values for trends *p* = 0.036, *p* = 0.809, *p* = 0.715 for Pb, Cd or Hg, respectively; ANOVA) (Table 2). However, it should be noted for Cd that the large number of women with levels below the limit of detection meant that there was an excess of individuals in quintile 1 (Table 2).

## 4 Discussion

We did not find any evidence of any effects of Pb, Cd or Hg levels on the secondary sex ratio in this study, with the exception of some evidence suggesting a decline in the ratio in the third quintile for Pb: this effect was not evident in the adjusted logistic model. The effect of Cd has not been studied before to our knowledge, and that of Hg is limited to a single study where exposure was likely to have been extremely high following industrial pollution, although not quantified [20]. In that study, there was a decline in the secondary sex ratio that was associated with an increase in male fetal deaths.

With regard to Pb, paternal occupational Pb exposure has been reported to cause an excess of female offspring [31,32]. It has been proposed that this excess is caused by low paternal testosterone and/or high gonadotropin levels at conception [33,34]. We were not able to measure paternal levels of Pb, Cd and Hg and so were unable to evaluate their association with the secondary sex ratio. However, maternal Pb levels might also be affected by the father's occupational exposure as a secondary effect, and this might provide an additional or perhaps alternative mechanism for the reported effect on the secondary sex ratio. Other studies have found no effect of paternal [35–37] or maternal [37] occupational exposure on the secondary sex ratio. To our knowledge, there has only been one previous systematic study of the direct effect of maternal blood Pb level on the secondary sex ratio [19]. The association was explored in a large study in Mexico, where there has been a dramatic decline in the sex ratio in the past 50 years. Concurrently, lead exposure in Mexico has been high due to the use of traditional Pb-glazed ceramics and leaded petrol. Several measures of Pb burden (maternal blood, cord blood, maternal tibia, maternal patella) were studied in 1980 pregnant women in 1994–1995 and 1997–2001. Using logistic regression models (adjusted for infants’ year of birth, maternal age and parity, but not paternal age) similar to the logistic model reported here, no consistent associations between any of the measures and the secondary sex ratio were found. This is in accordance with the finding in our study, although we measured maternal blood lead level only.

The strength of our study lies in the large number of participants that we were able to include. However, the number of participants is low with regard to the traditional determination of secondary sex ratios, which are usually done at a country or state level and include many thousands of individuals. A further strength is the measurement of blood levels of Pb, Cd and Hg in the first trimester of pregnancy: blood levels are likely to reflect recent exposure and the samples were taken close to both the time of conception and the likely time for early pregnancy loss. The results for Cd should be interpreted with caution as many of the results were below the limit of detection. For Pb, comparison with the study of Jarrell et al. [19] is limited by the greater exposure to Pb in their participants (median blood Pb in quintile 5 13.1 μg/dl vs 5.44 μg/dl in the present study). The mean levels of Pb, Cd and Hg may have been too low to show any effect, but they are broadly indicative of levels in similar European countries [26] and the secondary sex ratio has been reported as changing in European countries such as Denmark [4] and the Netherlands [3]. It is likely that there are many other environmental exposures and other factors influencing the sex ratio that were unable to account for in our models. Further work should be done in large cohorts in other countries to confirm our findings: the validity of further work could be strengthened with data on exposures to other chemicals that might also affect the secondary sex ratio and data on sex hormones during pregnancy.

## 5 Conclusion

We found no evidence for any effects of maternal levels of Pb, Cd or Hg on the secondary sex ratio in this study and conclude that it is unlikely that these metals make any significant contribution to the commonly observed decline in the secondary sex ratio.

## Funding

The UK Medical Research Council and the Wellcome Trust (Grant ref: 092731) and the University of Bristol provide core support for ALSPAC. The assays of the maternal blood samples were carried out at the Centers for Disease Control and Prevention (R. Jones) with funding from NOAA. C.M. Taylor was supported by a Daphne Jackson Trust Fellowship sponsored by the University of Bristol.

## Conflict of interest

There are no conflicts of interests to declare for any of the authors.

## Figures and Tables

**Table 1:** Categorical analysis of sex ratio according to maternal Pb, Cd and Hg levels during pregnancy in the ALSPAC study (1991–1992).

	Category		*p* value (chi-square test)
Maternal Pb (μg/dl)^a^	<5	≥5	
Male/female, *n* (% male)	1925/1778 (52.0)	326/303 (51.3)	
Secondary sex ratio	1.08	1.03	0.942
Maternal Cd (μg/l)^b^	<1	≥1	
Male/female, *n* (% male)	1811/1695 (51.6)	438/383 (53.3)	
Secondary sex ratio	1.07	1.14	0.381
Maternal Hg (μg/l)^c^	<5.8	≥5.8	
Male/female, *n* (% male)	2145/1992 (51.8)	23/17 (57.2)	
Secondary sex ratio	1.08	1.35	0.477
Maternal Hg (μg/l)^c^	<2.0	≥2.0	
Male/female, *n* (% male)	1213/1113 (52.1)	947/890 (51.6)	
Secondary sex ratio	1.09	1.06	0.701

Categories were chosen to reflect the available national guidelines [26].

a Categories for Pb were based on the US recommendation for the levels of concern in pregnancy [27,28].

b Categories for Cd were based on the German Federal Environmental Agency's levels of concern for adults ages 18–69 years [29].

c Two categories for Hg were analyzed: 5.8 μg/l is the level of concern recommended for adults by the US Environmental Protection Agency [30]; 2.0 μg/l is the level of concern for adults aged 18–69 years eating fish ≤3 times per month recommended by the German Federal Environmental Agency [29].

**Table 2:** Sex of infant and odds ratios for male child by quintiles of maternal Pb, Cd or Hg levels during pregnancy in the ALSPAC study (1991–1992).

	Quintiles of maternal blood level				
	1	2	3	4	5
*Maternal Pb*					
Male, *n* (%)	459 (53.1)	485 (55.5)	416 (48.1)	444 (51.2)	447 (51.8)
Secondary sex ratio	1.13	1.24	0.93	1.05	1.07
Maternal blood Pb (μg/dl), median (range)	2.11 (0.20–2.53)	2.82 (2.54–3.11)	3.42 (3.12–3.71)	4.13 (3.71–4.63)	5.90 (4.64–19.14)
*n*	865	879	868	870	868
Odds ratio for male child (95% CI)^a^	1 (reference)	1.04 (0.86, 1.42)	0.90 (0.70, 1.15)	1.01 (0.79, 1.30)	1.06 (0.82, 1.37)
*Maternal Cd*					
Male, *n* (%)	581 (52.3)	311 (52.0)	454 (50.9)	445 (52.4)	461 (53.6)
Secondary sex ratio	1.05	1.08	1.03	1.10	1.16
Maternal blood Cd (μg/l), median (range)	0.14 (0.14–0.14)	0.22 (0.20–0.24)	0.30 (0.25–0.37)	0.60 (0.38–0.95)	1.67 (0.96–6.30)
*n*	1135	598	895	856	867
Odds ratio of for male child (95% CI)^a^	1 (reference)	0.97 (0.76, 1.24)	1.08 (0.85, 1.37)	1.08 (0.85, 1.45)	1.11 (0.85, 1.45)
*Maternal Hg*					
Male, *n* (%)	427 (51.4)	448 (53.3)	434 (51.7)	439 (52.9)	422 (50.2)
Secondary sex ratio	1.06	1.15	1.07	1.12	1.01
Maternal blood Hg (μg/l), median (range)	0.95 (0.24–1.24)	1.44 (1.25–1.63)	1.86 (1.64–2.07)	2.36 (2.08–2.71)	3.73 (2.72–12.76)
*n*	836	840	844	835	841
Odds ratio for male child (95% CI)^a^	1 (reference)	1.00 (0.75, 1.31)	1.00 (0.76, 1.31)	0.99 (0.75, 1.30)	0.87 (0.66, 1.15)

a Logistic regression models adjusted for maternal and paternal age, and parity.
